# KSR-Based Medium Improves the Generation of High-Quality Mouse iPS Cells

**DOI:** 10.1371/journal.pone.0105309

**Published:** 2014-08-29

**Authors:** Kai Liu, Fang Wang, Xiaoying Ye, Lingling Wang, Jiao Yang, Jingzhuo Zhang, Lin Liu

**Affiliations:** State Key Laboratory of Medicinal Chemical Biology, College of Life Sciences, Nankai University, Tianjin, China; Muséum National d'Histoire Naturelle, France

## Abstract

Induced pluripotent stem (iPS) cells from somatic cells have great potential for regenerative medicine. The efficiency in generation of iPS cells has been significantly improved in recent years. However, the generation of high-quality iPS cells remains of high interest. Consistently, we demonstrate that knockout serum replacement (KSR)-based medium accelerates iPS cell induction and improves the quality of iPS cells, as confirmed by generation of chimeras and all iPS cell-derived offspring with germline transmission competency. Both alkaline phosphatase (AP) activity assay and expression of Nanog have been used to evaluate the efficiency of iPS cell induction and formation of ES/iPS cell colonies; however, appropriate expression of Nanog frequently indicates the quality of ES/iPS cells. Interestingly, whereas foetal bovine serum (FBS)-based media increase iPS cell colony formation, as revealed by AP activity, KSR-based media increase the frequency of iPS cell colony formation with Nanog expression. Furthermore, inhibition of MAPK/ERK by a specific inhibitor, PD0325901, in KSR- but not in FBS-based media significantly increases Nanog-GFP^+^ iPS cells. In contrast, addition of bFGF in KSR-based media decreases proportion of Nanog-GFP^+^ iPS cells. Remarkably, PD can rescue Nanog-GFP^+^ deficiency caused by bFGF. These data suggest that MAPK/ERK pathway influences high quality mouse iPS cells and that KSR- and PD-based media could enrich homogeneous authentic pluripotent stem cells.

## Introduction

iPS cells can be artificially produced from fibroblasts through the forced expression of Oct4, Sox2, Klf4, and c-Myc [1], [2]. Remarkably, mouse iPS cells are able to produce viable mice through tetraploid complementation [3], demonstrating their authentic pluripotency, and Tbx3 and Zscan4 further enhance their pluripotency [3], [4], [5]. Possible explanations for these findings could be that the stoichiometry of reprogramming factors strongly influences the epigenetic state and pluripotency of iPS cells [6].

Increasing evidence has shown that reprogramming efficiency of mouse iPS cells can be enhanced by addition of small molecules, such as BIX01294 (BIX, a G9a histone methyltransferase inhibitor) [7], valproic acid (VPA, a histone deacetylase [HDAC] inhibitor) [8], 5-azacytidine (AZA, a methyltransferase [DNMT] inhibitor) [8], [9], sodium butyrate (NAB, an HDAC inhibitor) [10] and vitamin C [11]. In addition, two signal pathway inhibitors, CHIR99021 (CH, a glycogen synthase kinase 3 beta [GSK3β] inhibitor) and PD0325901 (PD, a mitogen-activated protein kinase [MAPK]/extracellular signal-regulated kinase [ERK] inhibitor), were found to enhance completion and efficiency of reprogramming process [12]. Combination of two molecules (PD and CH, termed 2i) with leukaemia inhibitory factor (LIF) effectively maintains mouse ES cells in a naive state [13], [14]. Remarkably, mouse iPS cells can even be generated by a combination of small molecules without exogenes [15].

Small molecules have also been reported to enhance the efficiency and quality of human iPS cells. For instance, PD, CH, and SB431542 (SB, an anaplastic lymphoma kinase [ALK] inhibitor) [16] are frequently used in enhancing reprogramming. PD and CH are used to convert human pluripotent stem cells to the naive state [4], [17]. Combination of SB and PD, or SB, PD, and sodium butyrate (NAB) can convert partially reprogrammed colonies to a fully reprogrammed state, thereby improving the efficiency of reprogramming [18], [19]. Moreover, epigenetic modifier NAB is more reliable and efficient than VPA in generation of human iPS cells and contributes to more efficient reprogramming [20], [21].

Knockout serum replacement (KSR) facilitates generation of ES cells from embryos [22] and of viable iPS cell-derived mice by tetraploid embryo complementation [3]. Furthermore, use of KSR instead of fetal bovine serum (FBS) can greatly enhance the number of AP-positive colonies [23] and the pace and efficiency of Oct4-GFP expression during the reprogramming of iPS cells [24]. Whereas AP is activated early in the reprogramming process, expression of Nanog and Oct4 is only observed late in the process and marks fully reprogrammed cells [25]. Moreover, Nanog activation indicates that iPS cells have overcome reprogramming barriers [26]. Hence, Nanog activation is generally used to evaluate the quality of iPS cells.

However, the effectiveness of small molecules in FBS- and KSR-based media for generation of iPS cells and the differences between FBS and KSR in the derivation iPS cell lines have not yet been clearly defined. Additionally, the mechanisms underlying effects of FBS on iPS cell induction remain to be determined. In our study, we compared several media conditions for reprogramming mouse fibroblasts to iPS cells, and our data show that Nanog expression in iPS cells is greatly enhanced by KSR-based medium instead of FBS-based medium during reprogramming. Consistent with previous findings, KSR facilitates generation of all iPS cell-derived pups with germline transmission. Moreover, inhibition of MAPK by PD increases the efficiency and quality of iPS cells, as indicated by Nanog expression but not by AP activity.

## Materials and Methods

### Ethics Statement

The care and use of mice for this research were based on the protocols of the animal research guidelines approved by the Institutional Animal Care and Use Committee (IACUC) of Nankai University. Additionally, the IACUC of Nankai University approved our study.

### iPS cell generation

Mouse embryonic fibroblasts (MEFs) and adult fibroblasts were derived from C57BL/6J as previously described [27], [28]. Mice were sacrificed, and fibroblasts were isolated by washing, peeling, mincing, and culturing in MEF medium (DMEM containing 10% FBS) according to the protocol “Manipulating the Mouse Embryo” published by Cold Spring Harbor Laboratory. iPS cells were induced by transduction with four Yamanaka factors (pMXs-Sox2, Klf4, Oct4 and c-Myc) using a standard protocol [1], [29] and isolated based on morphological criteria and on Nanog-promoter green fluorescent protein (Nanog-GFP) expression. From day 0 to day 2 post-infection, cells were cultured in MEF medium. By day 3, approximately 10,000 infected fibroblasts were placed onto new feeder-coated dishes in basal mouse embryonic stem (ES) cell medium containing knockout Dulbecco' modified Eagle medium, 1000 U/ml LIF, 0.1 mM β-mercaptoethanol, 1 mM L-glutamine, 0.1 mM nonessential amino acids, 100 units/ml penicillin and 100 µg/ml streptomycin, supplemented with 20% FBS (named FBS medium) or with 20% KSR (named KSR medium) with or without small molecules. Small molecules were purchased from Stemgent or from Sigma and supplemented at following final concentrations: PD0325901 (PD, 1 µM), CHIR99021 (CH, 3 µM), SB431542 (SB, 2 µM), sodium butyrate (NAB, 0.25 mM), and bFGF (4/0.4 ng/ml, Gibco). Media were changed daily, and ES cell-like colonies picked and passaged using standard protocols.

### Alkaline phosphatase staining (AP) and immunofluorescence staining

AP activity was detected using an Alkaline Phosphatase Substrate Kit III (Vector, sk-5300), according to the instruction manual. AP-positive colonies under different conditions were counted and analysed by StatView software.

Immunofluorescence staining was performed as previously described [30]. Cells were washed in PBS, fixed in 3.7% paraformaldehyde, permeabilised with 0.1% Triton X-100, blocked with blocking solution, and incubated overnight at 4°C with primary antibodies Oct4 (1∶200, sc9081, Santa Cruz), Nanog (1∶200, AB80892, Abcam), or SSEA-1 (1∶200, MAB4301, Millipore). After wash with PBS for three times, cells were incubated with a secondary antibody (1∶200, Goat Anti-Rabbit IgG (H+L) Alexa Fluor 594 111-585-003 Jackson 1.5 mg or Goat Anti-Mouse IgG (H+L) FITC 115-095-003 Jackson 2 mg). Nuclei were stained using Vectashield medium (Vector) added with Hoechst 33342 (Sigma). Fluorescence images were captured using a Zeiss fluorescence microscope (AxionVision Z1).

### RNA extraction and quantitative real-time PCR (qPCR)

Total RNA was isolated from mouse ES cells, fibroblasts or iPS cells using an RNeasy Mini Kit (Qiagen) and reverse transcribed using M-MLV Reverse Transcriptase (Invitrogen). cDNA was used as a template for qPCR. qPCRs were performed with the FastStart Universal SYBR Green Master (Roche) according to manufacturer's instructions. Signals were detected with an iCycler iQ5 2.0 Standard Edition Optical System (Bio-Rad). The relative expression level of the target genes was normalised by β-actin or GAPDH, and calculated by ΔΔCt method. Primers were designed using the IDT DNA website or as previously described (see Table S1) [3]. All qPCRs were performed by more than three biological replicates, and the results indicated as means with error bars.

### Fluorescence-activated cell sorting (FACS) analysis

Flow cytometry was used to analyse Nanog-GFP percentage of fibroblasts and iPS cells using a BD LSR analyser (BD Biosciences), and data were calculated using CellQuest Pro or Flow Jo software. More than two independent experiments and negative controls were assayed to verify the percentage of GFP-positive cells.

### Four- to eight-cell embryo injection and chimera generation

Microinjection of ES/iPS cells into four- or eight-cell embryos, which is facilitated by a Piezo injector, can generate not only germline competent chimeras but also complete ES cell-derived mice with high efficiency [31], an alternative assay to both the tetraploid embryo complement and the injection of diploid blastocysts to test developmental pluripotency. Approximately 10–5 ES/iPS cells (C57BL/6, black) were injected into eight-cell embryos (ICR, white) as hosts using a Piezo injector as previously described. Injected embryos were cultured overnight in KSOM medium, and developed blastocysts were transferred into uterine horns of 2.5 day post coitum (dpc) surrogate mice. Pregnant females delivered pups naturally at approximately 19.5 dpc. The pups were identified by coat colour. Some chimeras and all iPS cell-derived pups were randomly selected for breeding with ICR and further examined for germline transmission.

### Teratoma formation and haematoxylin and eosin (HE) staining

Approximately 1×10^6^ iPS cells were subcutaneously injected into immunodeficient nude mice (SCID). After 1 month, mice were sacrificed to assess teratoma formation. Teratomas were excised, fixed in 3.7% paraformaldehyde, washed in 70% ethanol, embedded in paraffin, and sectioned for histological examination by HE staining.

### Embryoid body (EB) formation and differentiation

Mouse iPS cells were removed off feeder cells twice based on their differences in adherence to the bottom of dish. Then, cells were transferred to low-adhesive 35 mm non-coated plates and cultured in ES medium without LIF. Aggregated EBs were formed after 4–7 days and transferred onto gelatin-coated tissue culture dishes for differentiation for another 5–7 days. Differentiated cells were fixed for immunofluorescence staining using primary antibodies of three embryonic germ layers, including AFP (1∶1, DAKO, DAK-N150130, endoderm), SMA (1∶200, Abcam, ab5694-100, mesoderm), and βIII-tubulin (1∶200, Abcam, CBL412, ectoderm). The secondary antibodies and protocol were same to the method described for immunofluorescence staining and microscopy above.

### Statistical analysis

All the experiments were performed more than three times (n≥3), and the mean ± standard error (SE) or typical pictures were shown. Statistical analysis of means and variance were compared by Fisher's protected least-significant difference (PLSD) using StatView software from SAS Institute Inc (Cary, NC). Significant differences were defined as * (P<0.05) or ** (P<0.01).

## Results

### FBS impairs formation of high-quality iPS cells and KSR improves the quality of iPS cells, as determined by Nanog-GFP expression

We employed mouse embryonic fibroblasts (MEFs) carrying the Nanog-promoter green fluorescent protein (Nanog-GFP) and generated mouse iPS cells with four factors (*Oct4*, *Sox2*, *Klf4* and *c-Myc*). These cells were cultured in MEF medium until day 2 post-infection, and then 10,000 infected MEFs were separated and placed onto new feeder-coated dishes. Subsequently, MEF medium was replaced by different types of ES media on day 3, and the efficiencies of iPS cell induction were compared among these different induction media on day 12.

**Figure 1:**
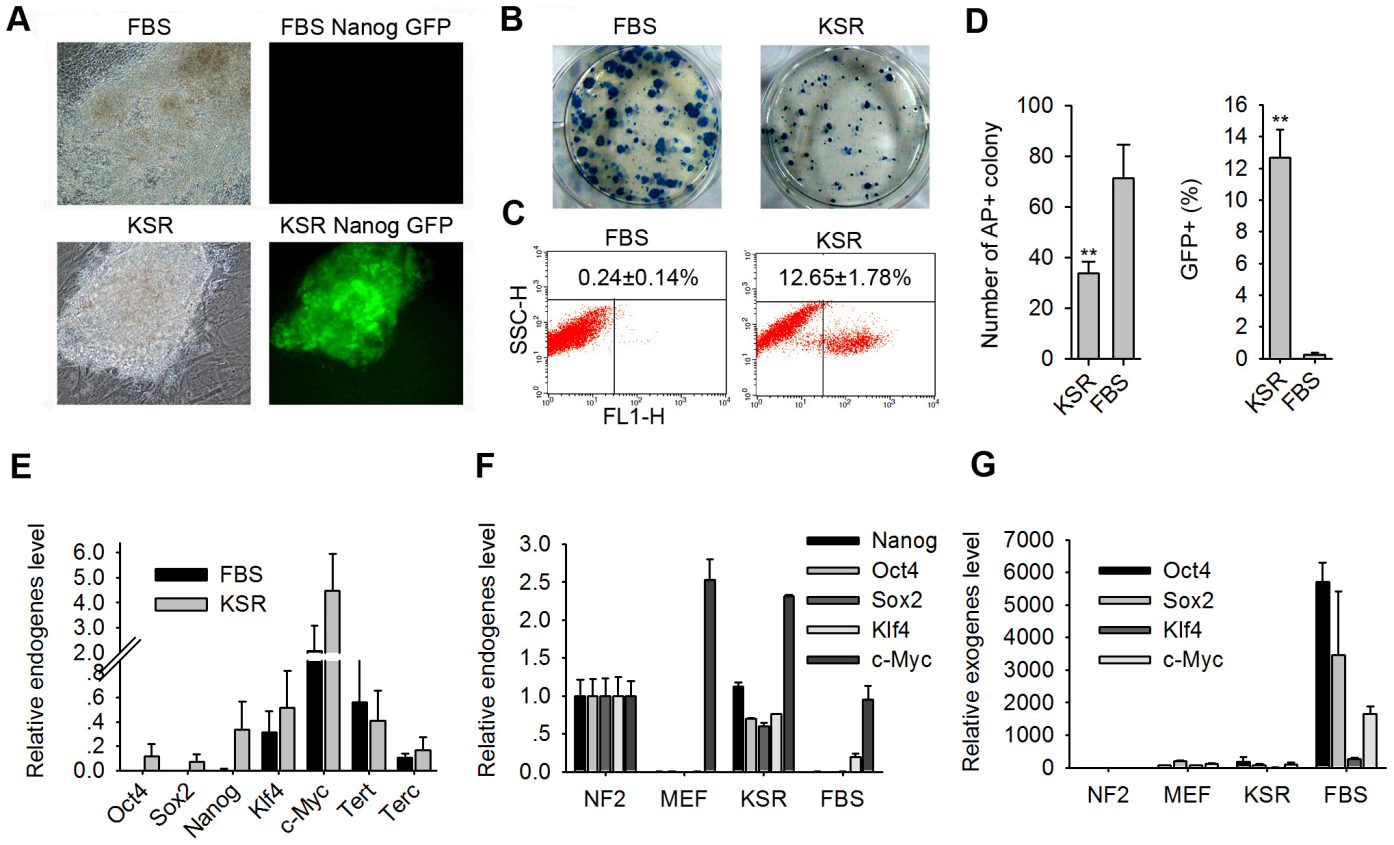
KSR improves the quality of iPS cells derived from MEFs. (A) Morphology and Nanog-GFP fluorescence in primary iPS cells on day 12 in the FBS or KSP induction media. (B–E) Comparison of primary iPS cells between FBS group and KSR group on day 12 using different assays, including the following: AP staining (B), Nanog-GFP^+^ FACS (C), statistical analysis of the AP^+^ colony number and of the percentage of Nanog-GFP^+^ cells (D), and endogene expression by qPCR (E). (F–G) qPCR analysis of expression of pluripotent endogenes and exogenes in iPS cell lines induced in KSR medium or in FBS medium at passage 5. Normal mouse ES cells (NF2) were used as a positive control, whereas MEFs used as a negative control.

First, we compared common mouse ES medium with 20% FBS and induction medium with 20% KSR for iPS cell colony formation. On day 12 post-infection, iPS cell colonies induced by KSR were generally more compact than those induced by FBS. Notably, most colonies grown in the KSR medium expressed Nanog-GFP, whereas few colonies in the FBS medium expressed Nanog-GFP (Fig. 1A).

FACS analysis was used to quantify Nanog-GFP-positive (Nanog-GFP^+^) cells on day 12. Consistently, the percentage of GFP^+^ cells grown in KSR based medium increased to 12.65%±1.78% (mean ± SE), whereas the percentage of GFP^+^ cells in FBS medium increased by only 0.24%±0.14% (Fig. 1C and 1D). Interestingly, contrary to the Nanog-GFP expression analysis, number of AP-positive (AP^+^) colonies was almost two times higher in FBS group (iPS cells generated in FBS medium) than in KSR group (iPS cells generated in KSR medium) (Fig. 1B and 1D).

Quantitative real-time PCR (qPCR) analysis showed that KSR group on day 12 expressed more endogenous pluripotent genes (*Oct4*, *Sox2*, *Nanog*) than did FBS group and that both groups expressed *Klf4*, *c-Myc* and telomerase-associated endogenes (*Tert*, *Terc*) (Fig. 1E).

### IPS cell lines induced by KSR show better quality than those induced by FBS at early passage

We carefully picked up compact iPS cell colonies on day 12 post-infection and changed the medium to normal mouse ES medium with FBS. Stable iPS cell lines were obtained for subsequent experiments. Endogenous and exogenous stem cell markers were examined by qPCR, and three different cell lines from KSR medium and from FBS medium at passage 5 (P5) were selected for further analysis. Expression levels of endogenous *Oct4*, *Sox2*, *Nanog*, *Klf4*, and *c-Myc* were much higher in KSR group at P5 than those levels in FBS group at P5 (Fig. 1F). Meanwhile, four exogenous transgenes were silenced more completely in KSR group at P5 than those of FBS group at P5 (Fig. 1G).

### Small molecules fail to rescue reduced generation of Nanog-GFP^+^ iPS cell clones caused by FBS

Next, we attempted to determine why FBS prevented Nanog-GFP^+^ colony formation and tested whether small molecules could rescue iPS cell deficiency by FBS. Four small molecules alone (PD, CH, SB, and NAB) or in combination (2i = PD+CH, 3i = PD+CH+SB, and 4i = PD+CH+SB+NAB) were used to rescue Nanog-GFP^+^ cells in FBS medium. We added small molecules from day 3 to day 12 in FBS medium. Subsequently, AP analysis and FACS analysis were performed on day 12 post-infection in various induction conditions.

**Figure 2:**
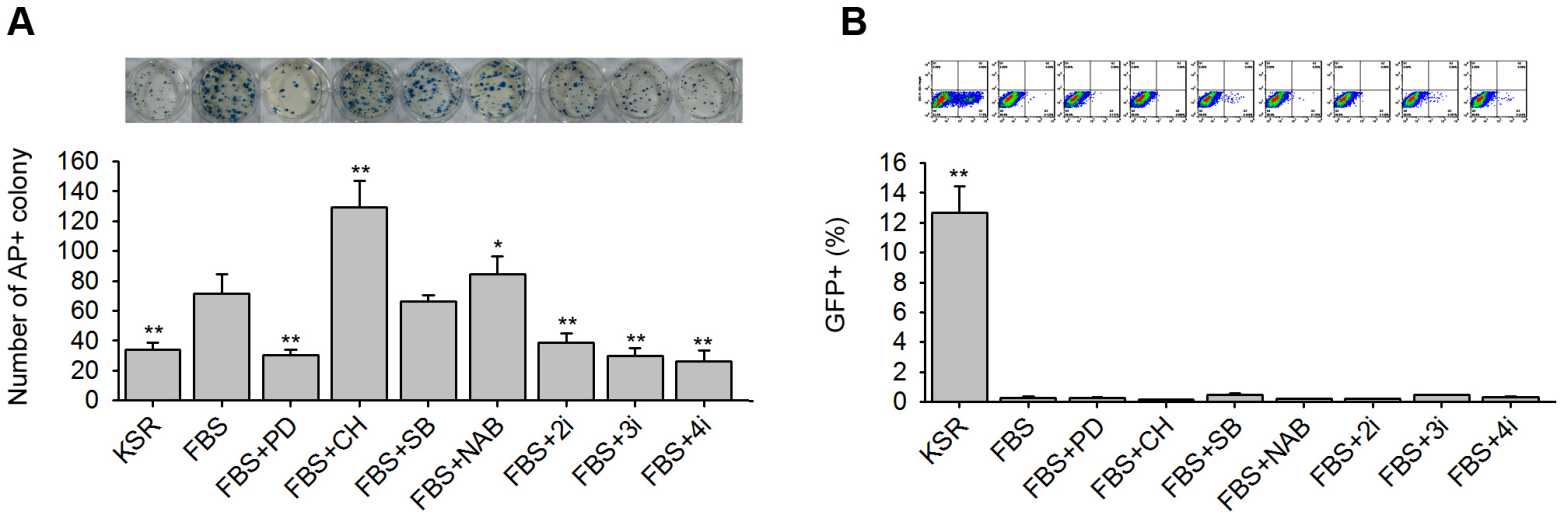
Effects of small molecules on iPS cell induction in FBS medium. (A) Representative AP staining pictures (upper) and quantitative analysis of AP^+^ colonies (lower) in different induction media on day 12; (B) Representative FACS images (upper) and quantitative analysis of Nanog-GFP^+^ cells in the same series media on day 12. Media series were the KSR medium, the FBS medium and FBS media with different small molecules (PD, CH, SB, NAB, 2i = PD+CH, 3i = PD+CH+SB, and 4i = PD+CH+SB+NAB, respectively).

Compared with the FBS medium alone, SB did not have a distinct influence on its AP^+^ colony number, and CH and NAB could improve their AP^+^ colony number. In contrast, PD, 2i, 3i, and 4i decreased the number of AP^+^ colonies (Fig. 2A). Compared with the KSR medium alone (nearly 13%) by FACS analysis, all small molecules tested could not distinctly improve the percentage of Nanog-GFP^+^ cells (less than 1%) (Fig. 2B).

### Inhibition of MAPK/ERK by PD in KSR improved Nanog-GFP^+^ iPS cells derived from MEFs

Nutrients in FBS medium were complex, and each lot of FBS was different. On the assumption that FBS may contain factors that promote differentiation of mouse ES cells, we focused next on iPS cell induction by KSR, which is a synthetic medium with little variable from lot to lot.

**Figure 3:**
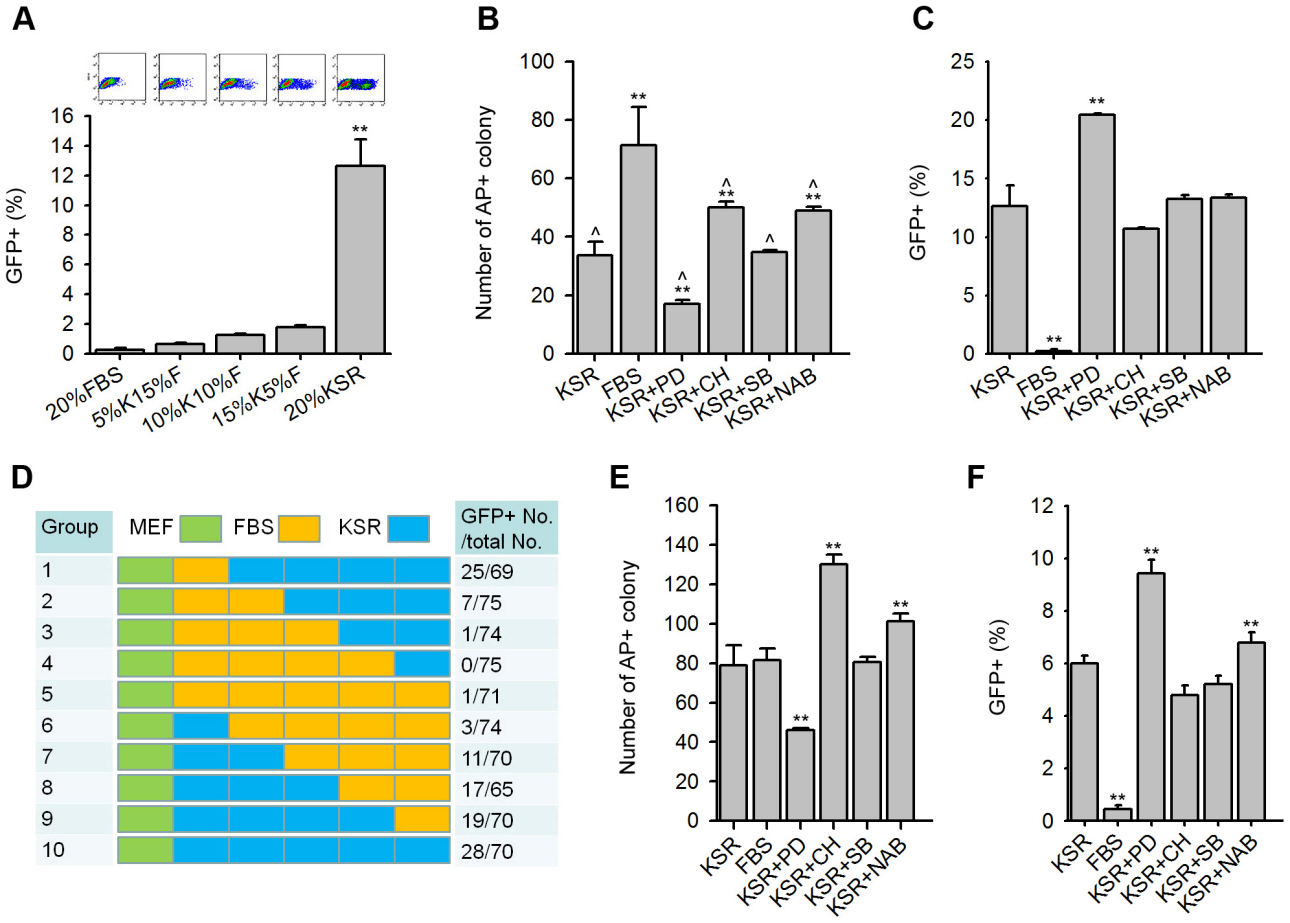
Effects of FBS and small molecules on reprogramming in KSR medium. (A–C) IPS cells derived from MEFs. Representative FACS images (upper) and the percentage of Nanog-GFP^+^ cells by FACS analysis (lower) in different FBS/KSR ratio media on day 12 post-infection (A). AP^+^ colony number (B) and the percentage of Nanog-GFP^+^ cells (C) in different induction media on day 12. Induction media were KSR, FBS, or KSR based media with different small molecules (PD, CH, SB, and NAB, respectively). (D–F) IPS cells derived from adult fibroblasts. Schematic of the induction process for 12 days in different media series. There were 10 groups with different series of culture media. Each box represented 2 day period using indicated medium (MEF medium: green, KSR medium: blue, FBS medium: yellow). On day 3, the same number of infected fibroblasts were replated on new dishes using the appointed media. Cells were monitored and counted on day 12 (right column was Nanog-GFP^+^ colony number/total colony number). The mean values were derived from three parallel experiments (D). AP^+^ colony number (E) and the percentage of Nanog-GFP^+^ cells (F) in different induction media on day 12 post-infection. ** represented significant differences between KSR and other conditions, ∧ represented significant differences between FBS and other conditions (P<0.01).

First, five different types of ES media with various proportions of KSR/FBS were used as induction media, followed by Nanog-GFP FACS analysis on day 12. Interestingly, compared with approximately 0.24%±0.14% GFP^+^ cells in 20% FBS, GFP^+^ cells increased to 0.64%±0.10%, 1.24%±0.10%, 1.78%±0.10%, and 12.65%±1.78% in 5% KSR+15% FBS, 10% KSR+10% FBS, 15% KSR+5% FBS, and 20% KSR, respectively (Fig. 3A). These results suggested that FBS inhibited Nanog-GFP^+^ iPS cell formation in a concentration-dependent manner.

Next, we examined the effect of small molecules in KSR medium. By AP analysis, compared with the KSR medium alone, SB did not have a distinct influence on the number of AP^+^ colonies, and CH and NAB could increase number of AP^+^ colonies. However, PD decreased the AP^+^ colonies. Nevertheless, number of AP^+^ colonies in the KSR medium supplemented with these small molecules was all less than that of FBS medium (Fig. 3B).

CH, SB, and NAB added in KSR did not have distinct influences on the percentage of Nanog-GFP^+^ iPS cells (approximately 12.6%) by FACS analysis. However, interestingly, PD enhanced Nanog-GFP^+^ cells by induction in KSR based medium by approximately 20% (Fig. 3C). These data indicate that PD, which inhibits MAPK/ERK pathway [13], [32], facilitated high-quality iPS cell formation with endogenous Nanog activation during reprogramming.

### PD in KSR improves Nanog-GFP^+^ iPS cells derived from adult fibroblasts

We also generated Nanog-GFP^+^ iPS cells from adult fibroblasts like generating iPS cells from MEFs. We divided the induction process (12 days) into six portions, with each for two days during reprogramming. During the first portion (day 1 and day 2 post-infection), adult fibroblasts were infected in MEF medium. Then, in part 2 to part 6 (day 3 to day 12), the same number of infected fibroblasts was replated on new dishes coated with feeder cells, and the MEF medium was substituted with different induction media in combinations (MEF medium: green, KSR medium: blue, and FBS medium: yellow) (Fig. 3D).

Nanog-GFP^+^ colony number/total colony number ratio was counted on day 12. Interestingly, the total colony number was close (approximately 70) among the different culture series. The ratios of Nanog-GFP^+^ colony number/total colony number were 25/69 in F2K8 (group 1: denoted by culture in FBS medium from day 3 to day 4, and then in KSR medium from day 5 to day 12), 7/75 in F4K6, 1/74 in F6K4, 0/75 in F8K2, 1/71 in F10, 3/74 in K2F8, 11/70 in K4F6, 17/65 in K6F4, 19/70 in K8F2, and 28/70 in K10 (Fig. 3D). These results suggest that treatment of KSR for longer time benefited Nanog-GFP activation.

Similarly, we efficiently obtained AP^+^ and Nanog-GFP^+^ iPS cell colonies from adult fibroblasts and compared effects of small molecules in KSR medium on day 12. SB did not have a distinct influence on the number of AP^+^ colonies; CH and NAB could increase the number of AP^+^ colonies, whereas PD still markedly decreased AP^+^ colonies (Fig. 3E). NAB slightly increased percentage of Nanog-GFP^+^ cells, and only PD could significantly enhance percentage of Nanog-GFP^+^ iPS cells on day 12 among all small molecules tested (Fig. 3F). These results further support the notion that PD in KSR improved formation of Nanog-positive iPS cells.

### IPS cell lines generated in KSR based medium can generate all iPS cell-derived pup with germline competency

Considering in potential clinical applications that adult fibroblasts are relatively easier to obtain from humans than embryonic fibroblasts, we next dissected the functional differences between stable iPS cell lines derived from mouse adult fibroblasts induced in FBS medium and those induced in KSR medium.

**Figure 4:**
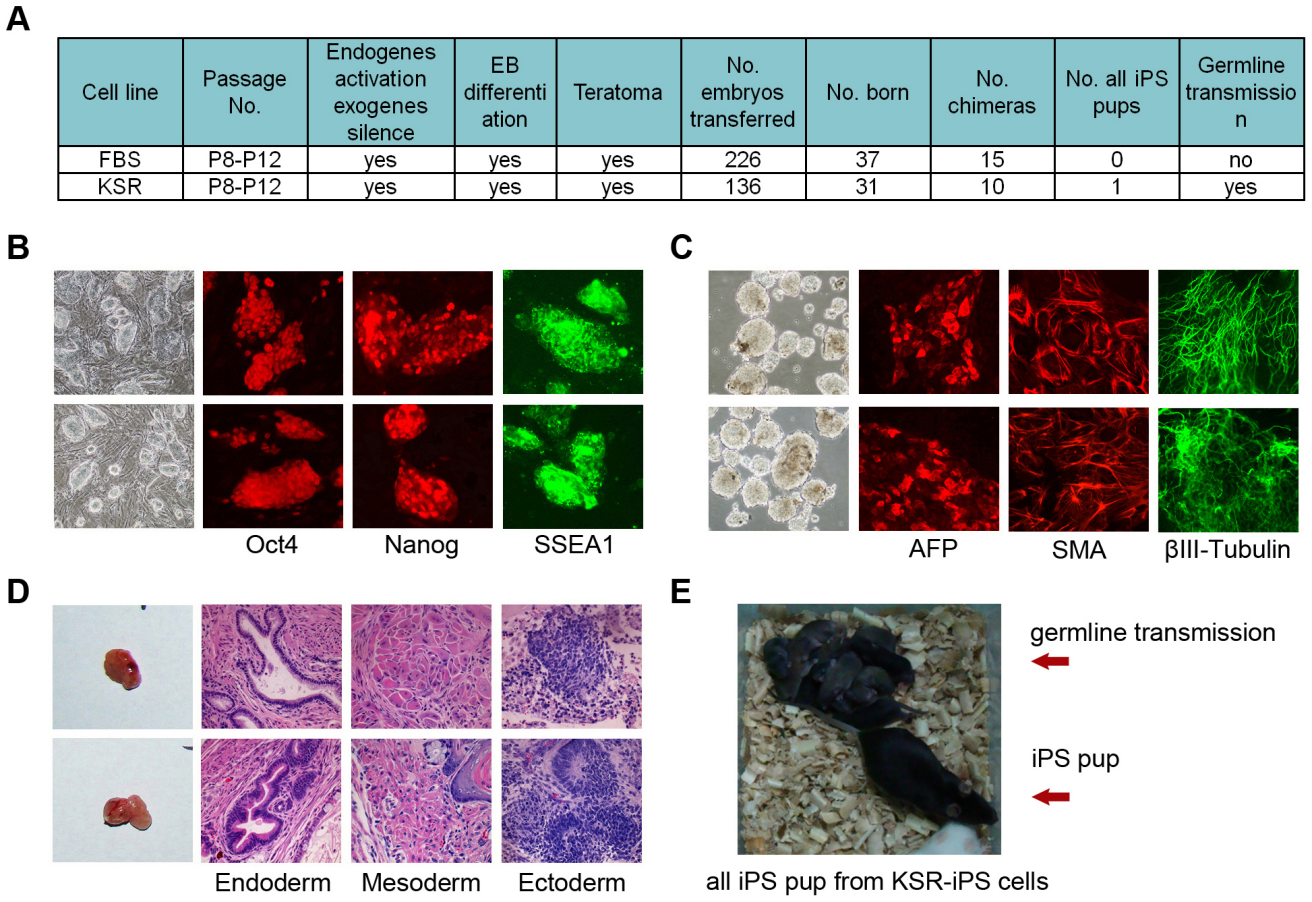
Comparison of pluripotency between iPS cell lines derived from adult fibroblasts under the induction condition of FBS or of KSR. (A) Summary table showing comparison of pluripotency between FBS group and KSR group at passage 12. (B–D) Characterisation and comparison of FBS group (upper) and KSR group (lower) at P12 by morphology and by immunofluorescence staining with ES markers (Oct4, Nanog and SSEA1) (B); EB formation and EB differentiation (endoderm marker AFP, mesoderm marker SMA, and ectoderm marker β-III-tubulin) (C); teratoma formation and HE staining of teratoma tissues: epithelium (endoderm), muscle (mesoderm), neural (ectoderm) (D). (E) All iPS cell-derived pup (black) with germline transmission produced from KSR group.

We chose two stable and good morphology iPS cell lines induced in the FBS medium at passage 12 (P12), also named WT iPS cell 5/6 in published papers [27], [28], [33], and two iPS cell lines induced in the KSR medium at P12, also named KSR iPS cell 1/2 in another published paper [33], to further analyse their pluripotency. Comparison for pluripotency between FBS and KSR group is summarized in Fig. 4A.

Both groups exhibited ES cell-like morphology and expressed Oct4, Nanog, SSEA1 (Fig. 4B), could efficiently form embryoid bodies (EB) and expressed markers for three germ layers (Fig. 4C). Moreover, both groups could steadily form teratomas with differentiation of clear three germ layers by histological analysis, following injection of iPS cells into SCID mice (Fig. 4D).

However, iPS cells from the FBS group and from the KSR group differed in chimeric mouse generation and in germline transmission capacity. Generation of offspring by microinjection of four- or eight-cell embryos provides a more stringent test of developmental pluripotency of iPS cells [31]. Each group had more than 100 eight-cell embryos injected to evaluate the efficiency in generating complete iPS cell-derived mice and chimeric mice. KSR group produced 10 chimeras and 1 all iPS cell-derived pup (identified by coat colour) when 136 embryos were transferred, whereas FBS group generated 15 chimeras without any all iPS cell-derived pups when 226 embryos were transferred (Fig. 4A). Moreover, the all iPS cell-derived pup produced from KSR group showed germline transmission competency (Fig 4E).

### Activation of the FGF pathway negatively affects authentic iPS cell enrichment

**Figure 5:**
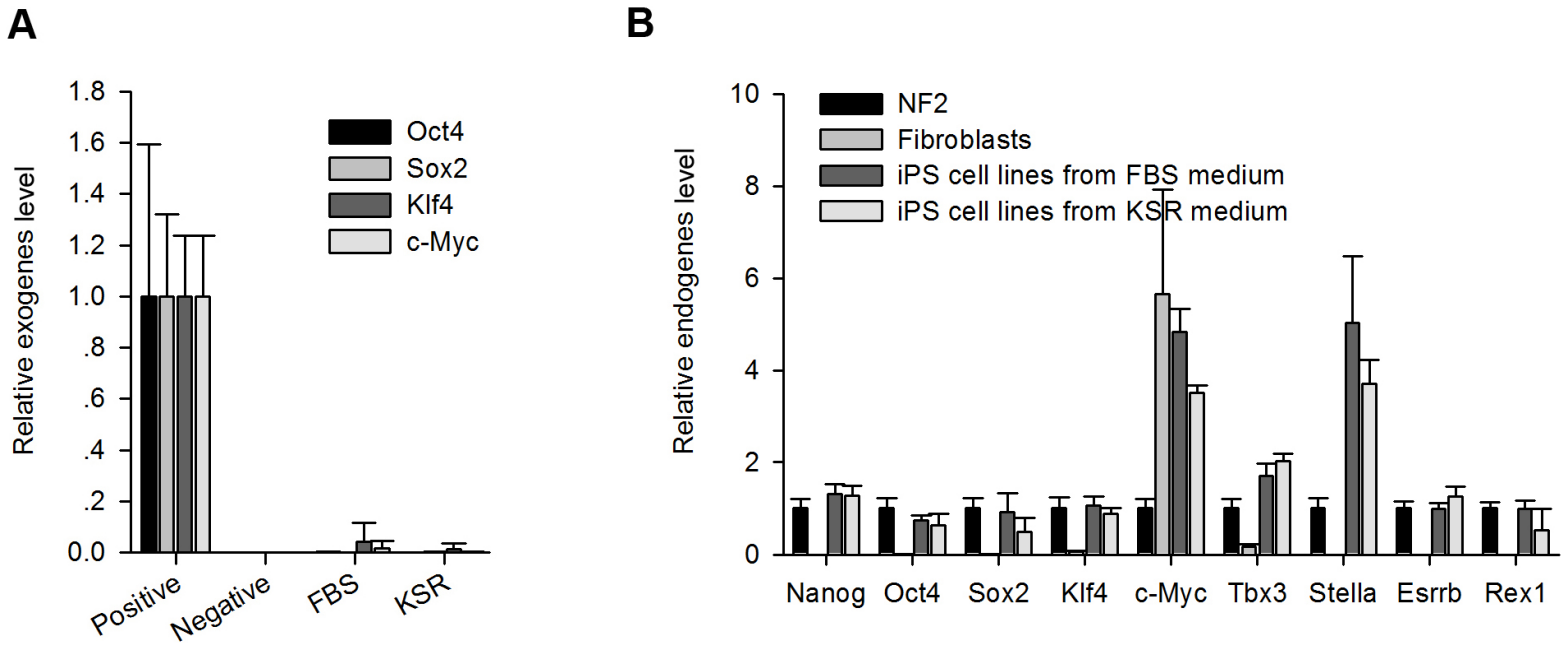
Expression of pluripotency genes of KSR group and FBS group at P12. (A) qPCR analysis of pluripotent exogenes (*Oct4*, *Sox2*, *Klf4*, *c-Myc*) in Positive (fibroblasts transfected by four Yamanaka factors), Negative (fibroblasts), FBS (iPS cell lines induced in the FBS medium at P12), and KSR (iPS cell lines induced in the medium at P12). (B) qPCR analysis of pluripotent endogenes (*Oct4*, *Sox2*, *Klf4*, *c-Myc*, *Nanog*, *Tbx3*, *Stella*, *Esrrb*, *Rex1*) in Positive (NF2, ES cells), Negative (fibroblasts), FBS (iPS cell lines induced in FBS medium at P12), and KSR (iPS cell lines induced in KSR medium at P12).

To determine the reason for the difference between these two groups, exogenous and endogenous pluripotent genes were analysed by qPCR. Neither exogenes (*Oct4*, *Sox2*, *Klf4*, *c-Myc*) (Fig. 5A) nor endogenes (*Oct4*, *Sox2*, *Klf4*, *c-Myc*, *Nanog*, *Tbx3*, *Stella*, *Esrrb*, *Rex1*) (Fig. 5B) showed significant differences in their expression levels between KSR and FBS group. Both groups displayed normal silencing of exogenes and activation of endogenes.

It is to note that C-Jun amino-terminal kinase (JNK), which is another MAPK pathway enzyme, negatively regulates reprogramming [34]. Thus, we hypothesised that some growth factors present in the serum, such as fibroblast growth factors (FGF), could be barrier to enrichment of high-quality mouse iPS cells [35]. Moreover, ERK can be activated by FGF [36] and inhibited by PD [13], [32].

**Figure 6:**
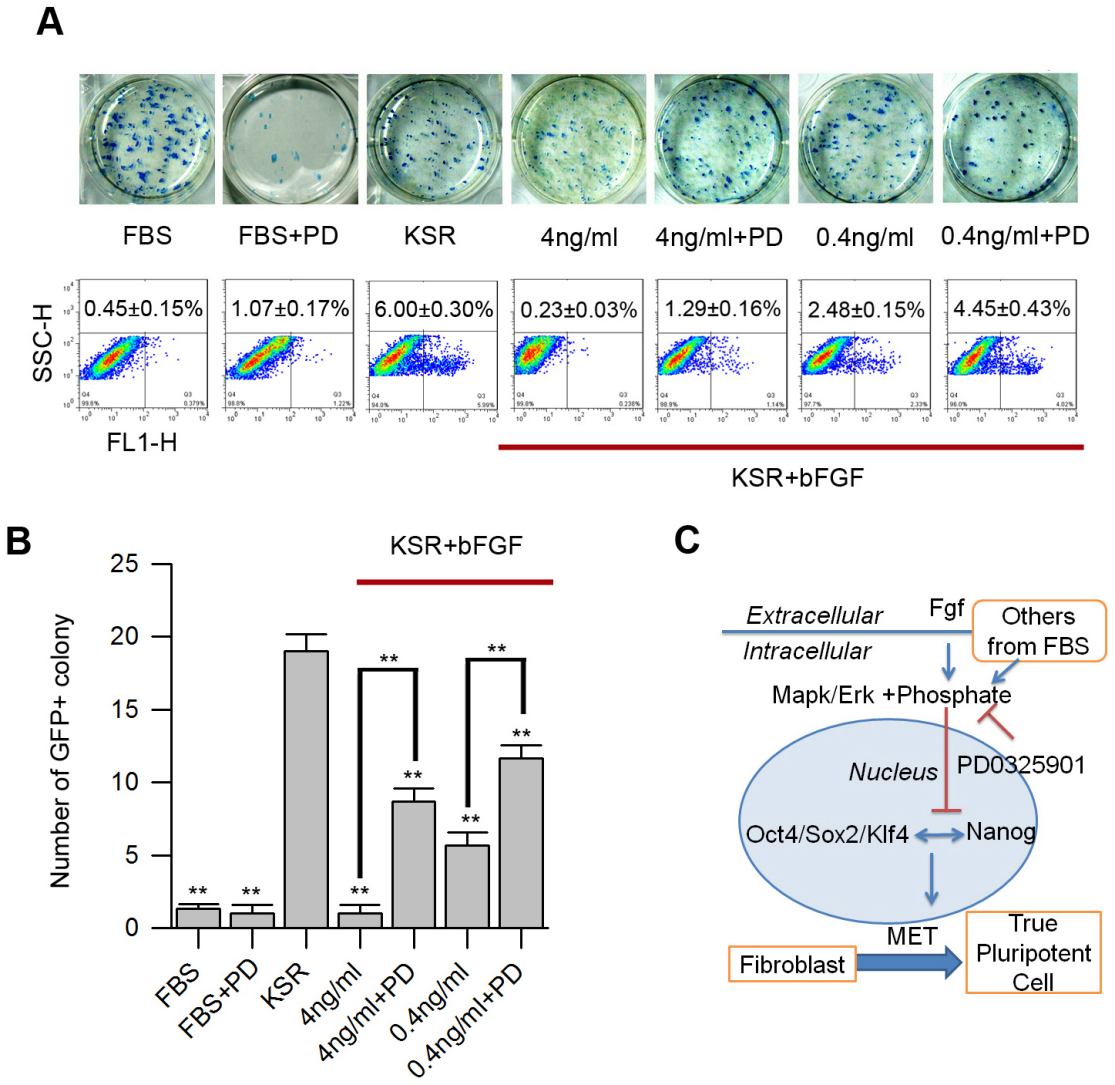
PD rescues repressive effect of bFGF in iPS cell induction. (A–B) Comparison of primary iPS cells derived from adult fibroblasts among different induction media on day 12 using different assays, including AP staining (A upper), the percentage of Nanog-GFP^+^ cells by FACS (A lower), and the number of Nanog-GFP^+^ colonies by fluorescence microscope (B). The induction media were FBS based and FBS with PD medium (FBS+PD); KSR medium and KSR medium with small molecules, including bFGF (4 ng/ml), bFGF (4 ng/ml) + PD, bFGF (0.4 ng/ml), and bFGF (0.4 ng/ml) + PD, respectively. (C) Functional model of MAPK/ERK pathway in the induction process. FGF may be one of the barriers in FBS that blocks formation of high-quality iPS cells. PD0325901, which selectively inhibits the MAPK/ERK pathway, benefits activation of Nanog and pluripotent endogenes during reprogramming.

Next, we chose bFGF, which is also called FGF2 [37], and PD to perform rescue experiments. AP assay, FACS, and count of Nanog-GFP^+^ colony numbers were employed to test effects of bFGF and PD in KSR medium during reprogramming. AP^+^ colonies in FBS medium were relatively larger and looser compared with iPS cell colonies in KSR medium; yet, the number of colonies did not differ between these two groups. bFGF (4 ng/ml) could loosen iPS cells when added in the KSR medium, whereas 4 ng/ml or 0.4 ng/ml bFGF did not influence on the number of AP^+^ colonies. PD caused the colonies to be compact and decreased the number of AP^+^ colony when added in FBS medium alone or when added with 0.4 ng/ml bFGF together in KSR medium (Fig. 6A and S2).

By FACS analysis of Nanog-GFP cells, Nanog-GFP^+^ colony numbers were detected on day 12. PD has significant positive effects and bFGF negative effects on the number of Nanog-GFP^+^ colonies. PD could partially increase the percentage of Nanog-GFP^+^ iPS cells in FBS medium by FACS assay, but we could not see an increase in Nanog-GFP^+^ colonies by fluorescence microscope because of the low fluorescence intensity (Fig. 4A and 4B). Similar to the effect of FBS in reprogramming, bFGF decreased the percentage of Nanog-GFP^+^ iPS cells, which correlated with concentrations when added in the KSR medium. Decline in the percentage of Nanog-GFP^+^ iPS cells caused by bFGF could be rescued by PD when added together with bFGF in KSR medium (Fig. 6A and 6B). Together, these results suggest that FGF was likely one of the inhibitory factors in FBS that influenced the quality of iPS cells.

## Discussion

Our data suggest that growth factors in the serum could decrease the enrichment of high-quality iPS cells during reprogramming. These data provide important insights into the optimisation of iPS cell induction media that could be used for enriching homogenous authentic pluripotent stem cells.

The purpose of our experiment was to rapidly and uniformly generate high-quality pluripotent iPS cell lines. We systematically tested the iPS cell quality on day 12 post-infection. Although the values of AP^+^ colonies were previously used [23], [24], our data showed the differences between AP^+^ and Nanog-GFP^+^ iPS cells. This suggests that the number of colony or AP staining is an inaccurate criterion to predict iPS cell quality and is variable among different donor cell derived iPS cells (Fig. 2 and 3), whereas Nanog activation is a good indicator of iPS cell quality [25]. We chose fibroblasts carrying GFP regulated by Nanog promoter as basic experimental material, and this model facilitated our next comparison of different induction media.

Instead of FBS, KSR produced high-quality iPS cell colonies (Fig. 1 and 4A), however we could pick stable and good iPS cell lines induced either in the FBS medium or in the KSR medium in our lab without significant differences in morphology, expression of pluripotent genes, EB formation, and teratoma formation (Fig. 4 and 5). Hence, both FBS and KSR can be used in generating good iPS cells, but KSR-based medium is beneficial for enriching authentic pluripotent stem cells.

Together with findings on the use of PD [13], [32], FGF [35], MAPK/ERK [36], networks in ES cells [38], and mesenchymal-to-epithelial transition (MET) during reprogramming [39], we outline a putative MAPK/ERK signal pathway during reprogramming (Fig. 6C). PD, which selectively binds and inhibits MEK and results in inhibition of phosphorylation and activation of MAPK/ERK [32] (Fig. S1), enhancing formation of high-quality iPS cells.

We found bFGF negatively affects Nanog-GFP^+^ iPS cell enrichment, whereas others report that bFGF improves reprogramming efficiency (AP positive number) in normal ES medium with serum [40], and bFGF improves iPS cell quality (Oct4-GFP^+^ colony number) in serum-free medium (named fSF1, containing KSR, bFGF, and N2) [41]. Interestingly, bFGF does not impair mouse ES cells to generate all ES cell-derived pups [17] and pluripotent state of mouse iPS cells [42], [43]; however, FGF signalling inhibition drives genome-wide demethylation to the epigenetic ground state of pluripotency of ES cells [44]. Thus, considering the other members of FGF family, such as FGF4 [45], [46], the effects of FGF and the MAPK/ERK pathway in reprogramming and in pluripotency maintenance still require further study.

We conclude that growth factors and MAPK/ERK present in the serum are barriers to reprogramming and that KSR and MAPK/ERK inhibition improves generating and enriching high-quality iPS cells.

## Supporting Information

Figure S1 **PD decreases relative protein level of pErk1/2 in mouse adult fibroblasts.** Western blotting analysis showed relative protein levels of pErk1/2 in mouse adult fibroblasts in different culturing media. Culturing media included FBS medium, KSR medium, FBS medium with PD (FBS+PD), and KSR medium with PD, respectively. Erk1/2 and β-actin served as loading control.
(DOC)

Figure S2 **PD decreases the number of AP^+^ colony.** Representative AP staining pictures and quantitative analysis of the AP^+^ colony number in different induction media on day 12. The induction media were FBS medium and FBS with PD medium (FBS+PD); KSR medium and KSR medium with small molecules, including bFGF (4 ng/ml), bFGF (4 ng/ml) + PD, bFGF (0.4 ng/ml), and bFGF (0.4 ng/ml) + PD, respectively. Arrow marked AP-negative colony.
(DOC)

Table S1 **Primers for qPCR analysis of endogenous and exogenous genes.**
(DOC)
