# Supplementary material for: KSR-Based Medium Improves the Generation of High-Quality Mouse iPS Cells
Source: PLoS One. 2014 Aug 29;9(8):e105309. doi: 10.1371/journal.pone.0105309 (PMC4149410; doi:10.1371/journal.pone.0105309)
Supplement: Figure S1 — PD decreases relative protein level of pErk1/2 in mouse adult fibroblasts. Western blotting analysis showed relative protein levels of pErk1/2 in mouse adult fibroblasts in different culturing media. Culturing media included FBS medium, KSR medium, FBS medium with PD (FBS+PD), and KSR medium with PD, respectively. Erk1/2 and β-actin served as loading control. (DOC) [file pone.0105309.s001.doc]

**Supporting Information**


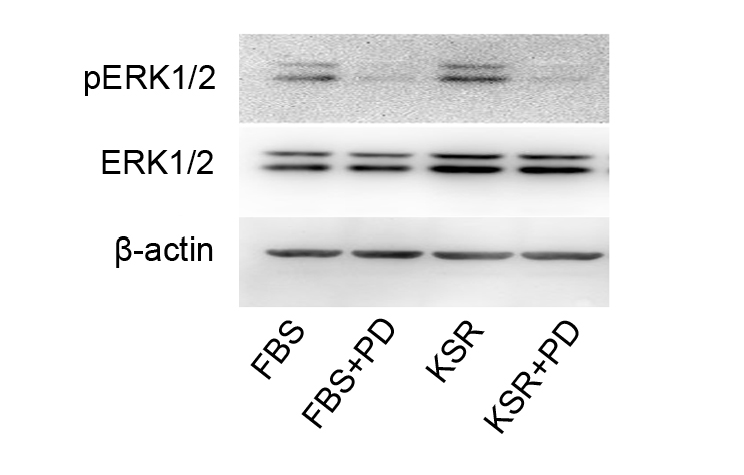


**Figure S1. PD decreased relative protein level of pErk1/2 in mouse adult fibroblasts.** Western blotting analysis showed relative protein levels of pErk1/2 in mouse adult fibroblasts in different culturing media. Culturing media included FBS medium, KSR medium, FBS medium with PD (FBS+PD), and KSR medium with PD, respectively. Erk1/2 and β-actin served as loading control.

**Materials and Methods**

Western blot

Western blot was performed on total cellular lysates using primary antibodies against Erk1/2 (sc-154, Santa Cruz, 1:1000), pErk1/2 (sc-7383, Santa Cruz, 1:1000), and β-actin (P30002, abmart, 1:1000). Cells were lysed in lysis buffer plus 1mM PMSF. Protein concentration was quantified by BCA Protein Assay Kit (23227 Thermo). The protein samples (20 μg) were separated by 10% SDS polyacrylamide gels and transferred to a PVDF membrane (0.2 μm Millipore ISEQ00010). After blocking with TBST buffer containing 5% nonfat dry milk, the membranes were incubated with the primary antibodies. After washing and incubation with secondary antibody (ECL anti-rabbit/mouse IgG, NA934/NA931 GE Healthcare, 1:5000), the proteins were detected using chemiluminescent HRP substrate reagent (WBKLS0100, Millipore).
