# Supplementary material for: KSR-Based Medium Improves the Generation of High-Quality Mouse iPS Cells
Source: PLoS One. 2014 Aug 29;9(8):e105309. doi: 10.1371/journal.pone.0105309 (PMC4149410; doi:10.1371/journal.pone.0105309)
Supplement: Figure S2 — PD decreases the number of AP+ colony. Representative AP staining pictures and quantitative analysis of the AP+ colony number in different induction media on day 12. The induction media were FBS medium and FBS with PD medium (FBS+PD); KSR medium and KSR medium with small molecules, including bFGF (4 ng/ml), bFGF (4 ng/ml) + PD, bFGF (0.4 ng/ml), and bFGF (0.4 ng/ml) + PD, respectively. Arrow marked AP-negative colony. (DOC) [file pone.0105309.s002.doc]

**Supporting Information**


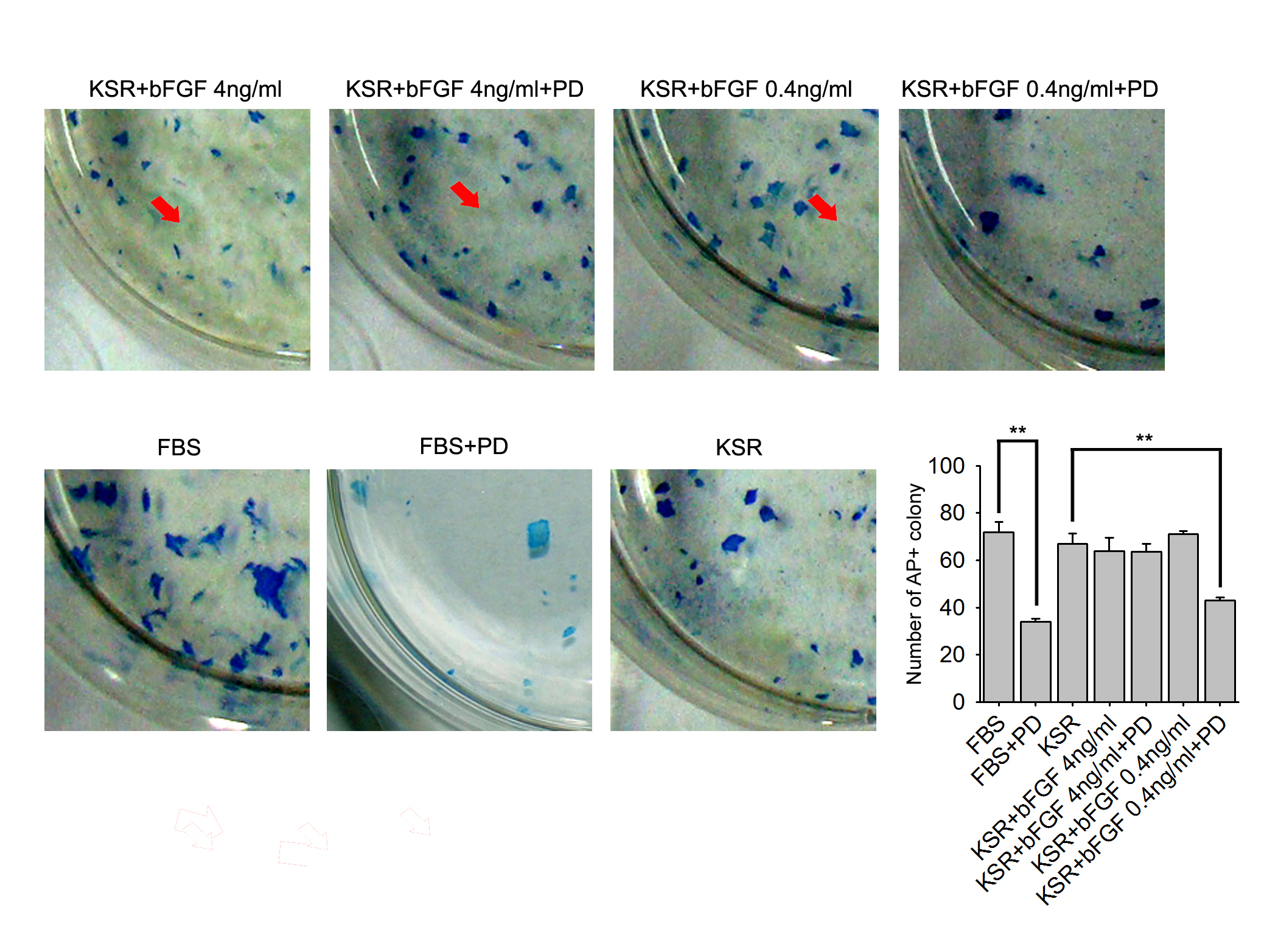


**Figure S2. PD decreased the number of AP^+^ colony.** Representative AP staining pictures and quantitative analysis of the AP^+^ colony number in different induction media on day 12. The induction media were FBS medium and FBS with PD medium (FBS+PD); KSR medium and KSR medium with small molecules, including bFGF (4 ng/ml), bFGF (4 ng/ml) + PD, bFGF (0.4 ng/ml), and bFGF (0.4 ng/ml) + PD, respectively. Arrow marked AP-negative colony.
