# Supplementary material for: KSR-Based Medium Improves the Generation of High-Quality Mouse iPS Cells
Source: PLoS One. 2014 Aug 29;9(8):e105309. doi: 10.1371/journal.pone.0105309 (PMC4149410; doi:10.1371/journal.pone.0105309)
Supplement: Table S1 — Primers for qPCR analysis of endogenous and exogenous genes. (DOC) [file pone.0105309.s003.doc]

**Supporting Information**

**Table S1. Primers for q**PCR analysis of endogenous and exogenous genes

| Genes name | Primer-Forward | Primer-Reverse | Genebank accession Number |
| --- | --- | --- | --- |
| ex-Oct4 | CCCAGTGTGGTGGTACGGGAAATC | AGTTGCTTTCCACTCGTGCT | * |
| ex-Sox2 | CCCAGTGTGGTGGTACGGGAAATC | TCTCGGTCTCGGACAAAAGT | * |
| ex-Klf4 | CCCAGTGTGGTGGTACGGGAAATC | GTCGTTGAACTCCTCGGTCT | * |
| ex-c-Myc | CCCAGTGTGGTGGTACGGGAAATC | GCTCGCTCTGCTGTTGCTGGTGATAG | * |
| endo-Oct4 | TCTTTCCACCAGGCCCCCGGCTC | TGCGGGCGGACATGGGGAGATCC | NM_013633 |
| endo-Sox2 | TAGAGCTAGACTCCGGGCGATGA | TTGCCTTAAACAAGACCACGAAA | NM_011443 |
| endo-Klf4 | CCATCGGACCTACTTATCTGC | AAAACCTCAAACCAAAACCC | NM_010637 |
| endo-c-Myc | TGACCTAACTCGAGGAGGAGCTGGAATC | AGTTTGAGGCAGTTAAAATTATGGCTGAAGC | NM_010849 |
| Nanog | CAGCCCTGATTCTTCTACCAG | AGAACACAGTCCGCATCTT | NM_028016 |
| Tbx3 | CCACCCGTTCCTCAATTTGAACAG | CGGAAGCCATTGATGGTAAAGCTG | NM_011535 |
| Stella | ATCCGGAGGGAAGTTCAAAG | TCCCGTTCAAACTCATTTCC | NM_139218 |
| Esrrb | CAAGAGAACCATTCAAGGCAACA | CATCCCCACTTTGAGGCATTT | NM_011934 |
| Rex1/Zfp42 | TGAAAGTGAGATTAGCCCCGAG | GTCCCATCCCCTTCAATAGCAC | NM_009556 |
| β-actin | GAGATTACTGCTCTGGCTCCTA | GGACTCATCGTACTCCTGCTTG | NM_007393 |
| GAPDH | TCAACAGCAACTCCCACTCTTCCA | ACCACCCTGTTGCTGTAGCCGTAT | NM_008084 |

*Primers for exogenous transcription factor primer were based on the plasmid.
